# Inhibition and counterinhibition of Surfacen, a clinical lung surfactant of natural origin

**DOI:** 10.1371/journal.pone.0204050

**Published:** 2018-09-20

**Authors:** Yuliannis Lugones, Odalys Blanco, Elena López-Rodríguez, Mercedes Echaide, Antonio Cruz, Jesús Pérez-Gil

**Affiliations:** 1 Dept. Biochemistry, Fac. of Biology, Complutense University, Madrid, Spain; 2 Centro Nacional de Sanidad Agropecuaria, Mayabeque, Cuba; 3 Research Institut “Hospital 12 de Octubre (imas12)”, Madrid, Spain; Hospital for Sick Children, CANADA

## Abstract

Inactivation of pulmonary surfactant by different components such as serum, cholesterol or meconium contributes to severe respiratory pathologies through destabilization and collapse of airspaces. Recent studies have analyzed in detail how the interfacial properties of natural surfactant purified from animal lungs are altered as a consequence of its exposure to serum proteins or meconium-mobilized cholesterol. It has been also demonstrated that pre-exposure of surfactant to polymers such as hyaluronic acid provides resistance to inactivation by multiple inhibitory agents. In the current work, we have extended these studies to the analysis of Surfacen, a clinical surfactant currently in use to rescue premature babies suffering or at risk of respiratory distress due to congenital lack of surfactant. This surfactant is also strongly inhibited by both meconium and serum when tested in the captive bubble surfactometer (CBS) under conditions mimicking respiratory dynamics. As it occurs with native surfactant, Surfacen is markedly protected from inhibition by pre-exposure to hyaluronic acid, confirming that clinical surfactants can be improved to treat pathologies associated with strongly deactivating contexts, such as those associated with lung injury and inflammation. Remarkably, we found that, under physiologically-mimicking conditions, a cholesterol-free clinical surfactant such as Surfacen is less susceptible to inhibition by cholesterol-mobilizing environments than cholesterol-containing natural surfactant, as a consequence of a markedly reduced susceptibility to incorporation of exogenous cholesterol.

## Introduction

Pulmonary surfactant is a complex mixture of lipids and specific proteins. It is synthesized and secreted into the alveolar spaces by type II pneumocytes and its main function is to reduce surface tension at the respiratory air–liquid interface, avoiding alveolar collapse and reducing the work of breathing [1]. Therapeutic application of surfactant preparations to babies suffering of Newborn Respiratory Distress Syndrome (NRDS) has proven to be efficient, a fact that has been confirmed in a considerable number of clinical trials, so that exogenous surfactant therapy is a routine practice in patients with NRDS [2].

Adequate surfactant activity remains critical for optimal lung function throughout life, and secondary surfactant dysfunction or inactivation may contribute to the pathogenesis of severe lung diseases, including Acute Respiratory Distress Syndrome (ARDS) [3, 4] and Meconium Aspiration Syndrome (MAS)[5] among others. The incidence of acute lung injury (ALI) and ARDS in adults has been estimated in the order of 200,000 patients per year in the United States, with a mortality of near 40% [6, 7]. The clinical disorders associated with development of ARDS include sepsis, pneumonia, aspiration of gastric contents and major trauma. MAS remains a significant cause of morbidity and mortality in term newborn infants, despite improvements in perinatal management [8].

Surfactant inhibition, or inactivation, refers to processes that decrease or abolish the normal interfacial activity of pulmonary surfactant. Such processes may interfere with the adsorption of surfactant to form a phospholipid-rich functional surfactant film, preventing the film from reaching low enough surface tension upon compression, or affecting film re-spreading during expansion. A number of substances have been reported to inhibit pulmonary surfactant. The major inhibitory factors include plasma and serum proteins, lysophospholipids, free fatty acids, meconium (fetal feces expelled during stress), and supraphysiological levels of cholesterol [9]. Several investigations have explored the molecular mechanisms behind these inactivation processes as well as different strategies to protect and/or revert the inactivation of pulmonary surfactant [10]. The most promising results so far have reported that some non-ionic or ionic polymers (including dextran, polyethylene glycol (PEG), hyaluronan (HA), or chitosan) enhance the surface activity of clinical surfactants [11–18], native surfactant [19, 20] or different mixtures of synthetic phospholipids and surfactant proteins [21] in the presence of inactivating substances.

In the particular case of inhibition by cholesterol, many studies have been conducted to elucidate the role played by natural proportions of this lipid in the surfactant system while abnormally high proportions of cholesterol produce deleterious effects [22–26]. As a matter of fact, the balance between possible benefits of a limited proportion of cholesterol to sustain proper dynamics of surfactant membranes and films and the susceptibility to inhibition in pathogenic contexts is still a matter of debate. It has been suggested that the inhibitory effects of cholesterol could be associated with the exposure of surfactant to cholesterol in the presence of cholesterol-mobilizing agents, such as bile salts or cyclodextrin [27]. However, how much the nature of the surfactant preparation (in terms of composition, structure, etc) influences the eventual incorporation of cholesterol and its subsequent inhibitory effect has not been properly explored.

Recently, we have optimized protocols to assess lung surfactant inhibition by serum, cholesterol or meconium under physiologically-mimicking conditions in the captive bubble surfactometer (CBS)[19]. In the current study, we have used this approach to analyze the ability of serum and meconium to affect the biophysical properties of Surfacen, a clinical surfactant of porcine origin currently in use to treat premature babies suffering or at risk of RDS in Cuban hospitals. We have analyzed how much the susceptibility of Surfacen to inhibition is associated with incorporation of cholesterol and whether the exposure of this clinical surfactant to hyaluronic acid protects or reverts inhibition of Surfacen by either substance.

## Materials and methods

### Materials

Preparations of Surfacen, a clinical surfactant used in the NRDS therapy in Cuban hospitals [28], were obtained from the *Centro Nacional de Sanidad Agropecuaria* (CENSA, Mayabeque, Cuba). Surfacen is obtained from organic extracts of porcine bronchoalveolar lavages, which are subjected to acetone precipitation to reduce their content of neutral lipids. It is provided as a sterile white lyophilized powder, dosed in vials containing 50 mg phospholipid, 0.3 to 0.7 mg of hydrophobic proteins and 2–3 mg of other lipids. Each vial also contains 18 mg of sodium chloride of the original saline solution used to obtain the lavage [29, 30]. To reconstitute an aqueous suspension of Surfacen, the proper amount of the surfactant dry powder is weighed and suspended in distilled water, with 60 min incubation of the suspension at 37°C, with occasional vortexing, shortly before running functional experiments.

Native porcine lung surfactant was obtained from bronchoalveolar lavage of porcine adult fresh lungs obtained from the slaughterhouse, as previously described [31], and stored at −70°C until its use. The lungs were extracted from animals destined to human consumption and no animals were sacrificed for the sole purpose of these experiments. Bronchoalveolar lavage (BAL) was performed in porcine lungs with a NaCl 0.9% solution that was then centrifuged at 1.000xg for 5 min to remove cells and cell debris. BALs were stored at -20°C until use. To obtain surfactant complexes, we first performed a centrifugation at 100.000xg, 4°C, 1h to pellet the full membrane fraction, and these pellets were later centrifuged in a discontinuous density gradient for 2h, 120.000xg, 4°C. Solutions used to prepare the density gradient included NaBr 16% NaCl 0.9% for the higher density, NaBr 13% NaCl 0.9% for the intermediate density and NaCl 0.9% for the lower density layers. At the end of the density gradient centrifugation, surfactant complexes comprising lipids and all the interacting proteins, are concentrated as a compact layer between the last two density solutions. This material is composed of 90% lipids and around 10% proteins although only 6–8% by mass are specific surfactant-associated proteins (SP-A, -B and–C). About 80–85% by weight of the lipids are phospholipids, including around 75% phosphatidylcholine, 10–15% phosphatidylglycerol plus phosphatidylinositol and less than 5% phosphatidylserine and sphingomyelin. Almost half content of surfactant phosphatidylcholine fraction is dipalmitoylphosphatidylcholine (DPPC). Cholesterol is the main neutral lipid in this natural surfactant preparation, representing 5–10% by mass of total lipid mass [29].

First-passed meconium was a gift from Prof. H. W. Taeusch, from San Francisco General Hospital, and was obtained from urine-free diapers of three normal full term infants born at the hospital. The meconium was refrigerated, well-mixed and lyophilized before use. The use of this discarded human material for research had been approved by the San Francisco General Hospital Institutional Review Board overseeing clinical research, insofar as no commercial use is intended and no patient identifiers are used. Hyaluronic acid (HA, 120k) was obtained from Sigma (St. Louis, MO). Dry weight of meconium or HA was used to standardize mixtures with Surfacen. Final concentration of Surfacen was 20 mg/ml, for meconium 10 mg/mL and 0.25% for HA; before starting functional tests Surfacen and mixtures were incubated 30 min at 37°C in a thermomixer. Porcine serum was obtained from blood and used at a protein concentration of 100 mg/mL. Cholesterol was obtained from Avanti Polar Lipids (Birmingham, AL), and water-soluble cholesterol (complexed with methyl-β-cyclodextrine (MβCD)) was obtained from Sigma (C4951, St. Louis, MO).

The organic extract of Surfacen was obtained by chloroform/methanol extraction of Surfacen aqueous suspension. Supplementation of organic extract of Surfacen with cholesterol was achieved by adding the proper amount of cholesterol dissolved in chloroform to the organic extract of Surfacen before drying. The mixture was then dried under nitrogen and then in UNIVAP for 2 h to remove traces of solvent and later re-hydrated in saline solution for 60 min at 37°C with periodical stirring at 550 rpm every 10 min.

### Methods

Surfactant performance was evaluated at 37°C was evaluated by captive bubble surfactometry as described previously [32]. The chamber contained 5mM Tris-HCl pH 7, 150mM NaCl and 10% sucrose. After a small air bubble (0.035–0.040 cm^3^) was formed, approximately 150nL of surfactant (20 mg/mL) were deposited below the bubble surface. Such small volume was geometrically measured at the image from the microscope of the CBS, taking into account the height of the column of surfactant applied within a transparent capillary of known diameter. Following surfactant application, the change in surface tension (γ) was monitored over five minutes from the change in shape of the bubble (17). The chamber was then sealed and the bubble was rapidly (1s) expanded to 0.15cm^3^, to record post-expansion adsorption. Five minutes after expansion, quasi-static cycles started, where the bubble size was first reduced (by a 20% of its previous volume) and then enlarged in a stepwise fashion. There was 1 min inter-cycle delay between each of four quasi-static cycles and a further 1 min delay before starting dynamic cycles, in which the bubble size was continuously varied at 20 cycles/min. Data from initial and post-expansion adsorption are presented as averages from several experiments (n>3), while graphs plotting quasi-static (Q-static) and dynamic cycles correspond to single representative experiments. In order to study the inhibitory effect of serum, with a inhibitory mechanism that depends on the competence with surfactant for adsorption at the interface, we first injected 2 μL of full serum (~100 mg/mL protein concentration) to form a layer at the bubble air-liquid interface, and then surfactant was injected in the subphase near the surface [19]. To study the inhibitory effects of meconium or cholesterol, which typically act by perturbing composition and structure of surfactant membranes, surfactant was first premixed with cholesterol or meconium, and the mixtures were later injected into the subphase near the bubble. We used a concentration of surfactant of 20 mg/mL in all functional experiments. In some experiments, HA was premixed with surfactant or surfactant/meconium mixtures and immediately injected into the subphase as described above (19).

To determine the percentage of incorporation of cholesterol to the surfactants, the proper amount of surfactant was exposed to different proportions of cholesterol-loaded methyl-β-cyclodextrine (MβCD), incubated during 30 min, 550 rpm at 37 ^o^C in thermomixer. After spinning at 14800 rpm the pellet was resuspended in buffer and used to quantitate phospholipid content, using the phosphorus assay described elsewhere [33], and cholesterol concentration with an enzymatic colorimetric method commercially provided by Spinreact (Girona, Spain). Alternatively, incorporated cholesterol was quantitated in preparations resuspended from mixtures of surfactant organic extract and chloroform organic solutions.

Most figures represent the mean ± S.D. after averaging data from several (n>3) independent experiments. Typically, four different experiments have been repeated with each sample, except for experiments exposing surfactant to meconium, which have been repeated 9 times to document the high intrinsic variability of meconium effects. CBS compression-expansion experiments are shown as representative isotherms. To identify statistically significant differences, one-way ANOVA with Duncan post t-test analysis was performed (InfoStat 2018) [34]. Probability values of p<0.05 were considered significant.

## Results

**Fig 1:**
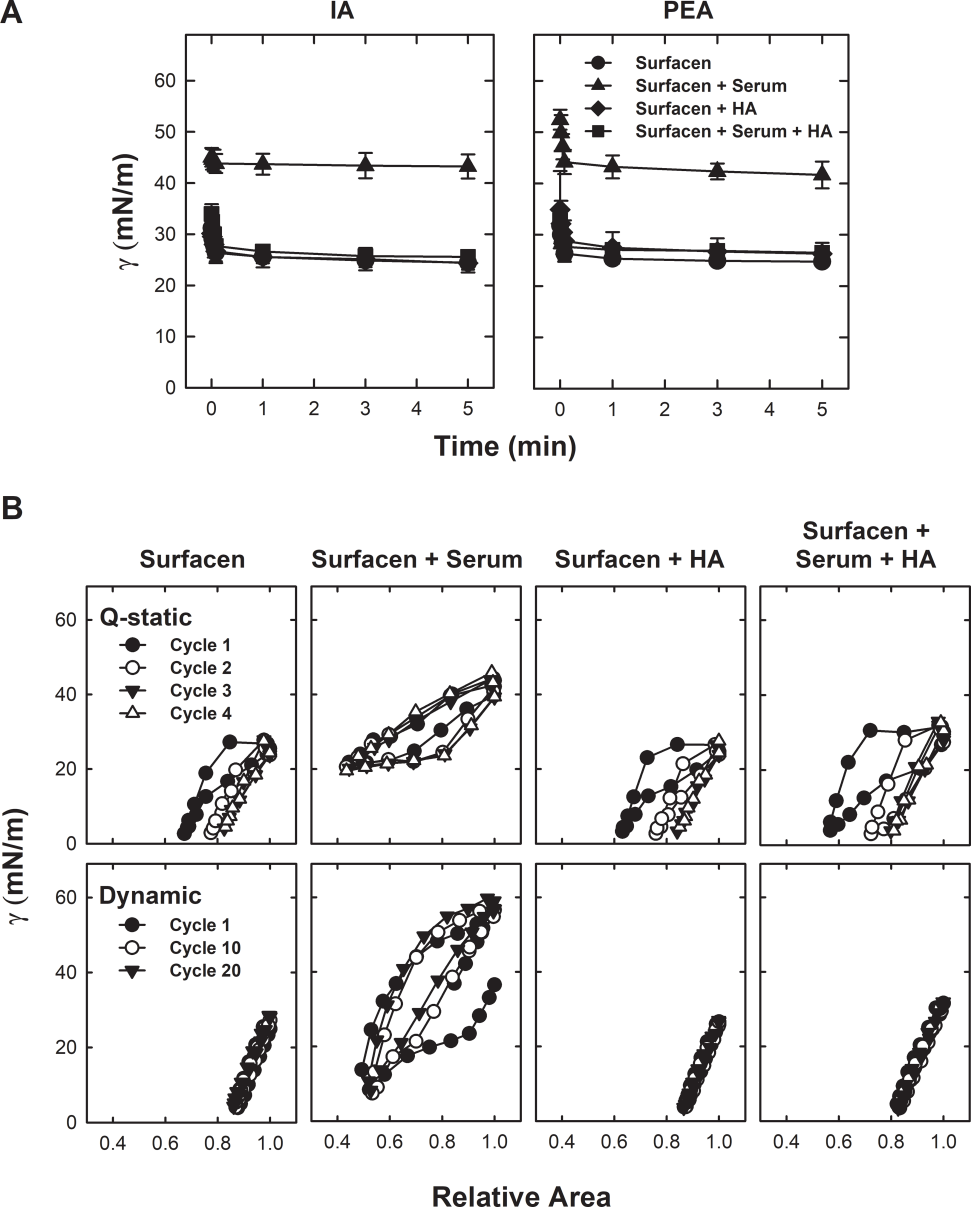
Inhibition of Surfacen by serum and its reversion by HA. Interfacial adsorption (A) and compression-expansion (B) isotherms of Surfacen films in the absence or presence of serum and/or HA. Data are means ± standard deviation in IA and PEA plots. A representative experiment is shown in each panel of compression-expansion isotherms, from the different repetitions (n>3) performed with each sample.

Fig 1A illustrates how Surfacen in the absence of serum rapidly forms a surface film that reduces surface tension to 24.4±1.4 mN/m after 5 min of initial adsorption and after expanding the bubble interface for 5 minutes. During the initial adsorption, Surfacen is able to reach the interface in a few seconds. When Surfacen was applied underneath a serum layer adjacent to the air-liquid interface, no adsorption of Surfacen occurred because the surface tension remained at 43.2±2.3 mN/m, close to the tension of a pure serum film, and significantly higher (p = 0.00001) than that produced by Surfacen in the absence of serum. After expansion, the minimal surface tension after 5 min also remained unchanged at 41.7±2.6 mN/m, indicating that Surfacen was not able to compete efficiently with serum components to reach the interface. In contrast, application of Surfacen pre-exposed to HA restored the ability of surfactant to reach the interface and lower surface tension to values of 25.6±0.6 mN/m even in the presence of serum (no significant difference with respect to Surfacen alone in the absence of serum). In principle, HA does not affect the composition and behavior of Surfacen surface film, as the equilibrium tension reached by Surfacen alone was similar to that of Surfacen pre-mixed with HA.

Fig 1B summarizes compression-expansion isotherms of Surfacen films formed in the absence or presence of serum. In the absence of serum, Surfacen cycling isotherms exhibited optimal behavior, with the first quasi-static cycles showing greater hysteresis that subsequent cycles. At the end of the fourth quasi-static cycle, surface tension dropped to 3.0±0.8 mN/m after an area reduction of less than 20% of the original area. Dynamic cycles show normal function of Surfacen in the absence of serum, with very low surface tension (3.1±0.7 mN/m) reached upon less than 20% area reduction. Surfacen (20 mg/mL) applied just below the surface of the bubble, underneath a pre-formed serum layer, was not able to lower surface tension below 20.6±1.6 mN/m (Q-static cycles) or 13.9±5.8 mN/m (dynamic cycles), values that were both significantly higher (p = 0.0001) than those reached in the absence of serum, even after as much as a 50% reduction of surface area. However, application of Surfacen pre-mixed with HA was able to restore the ability of Surfacen to get a minimum surface tension that was statistically indistinguishable from that reached by Surfacen in the absence of serum during both Q-static and dynamic cycles.

**Fig 2:**
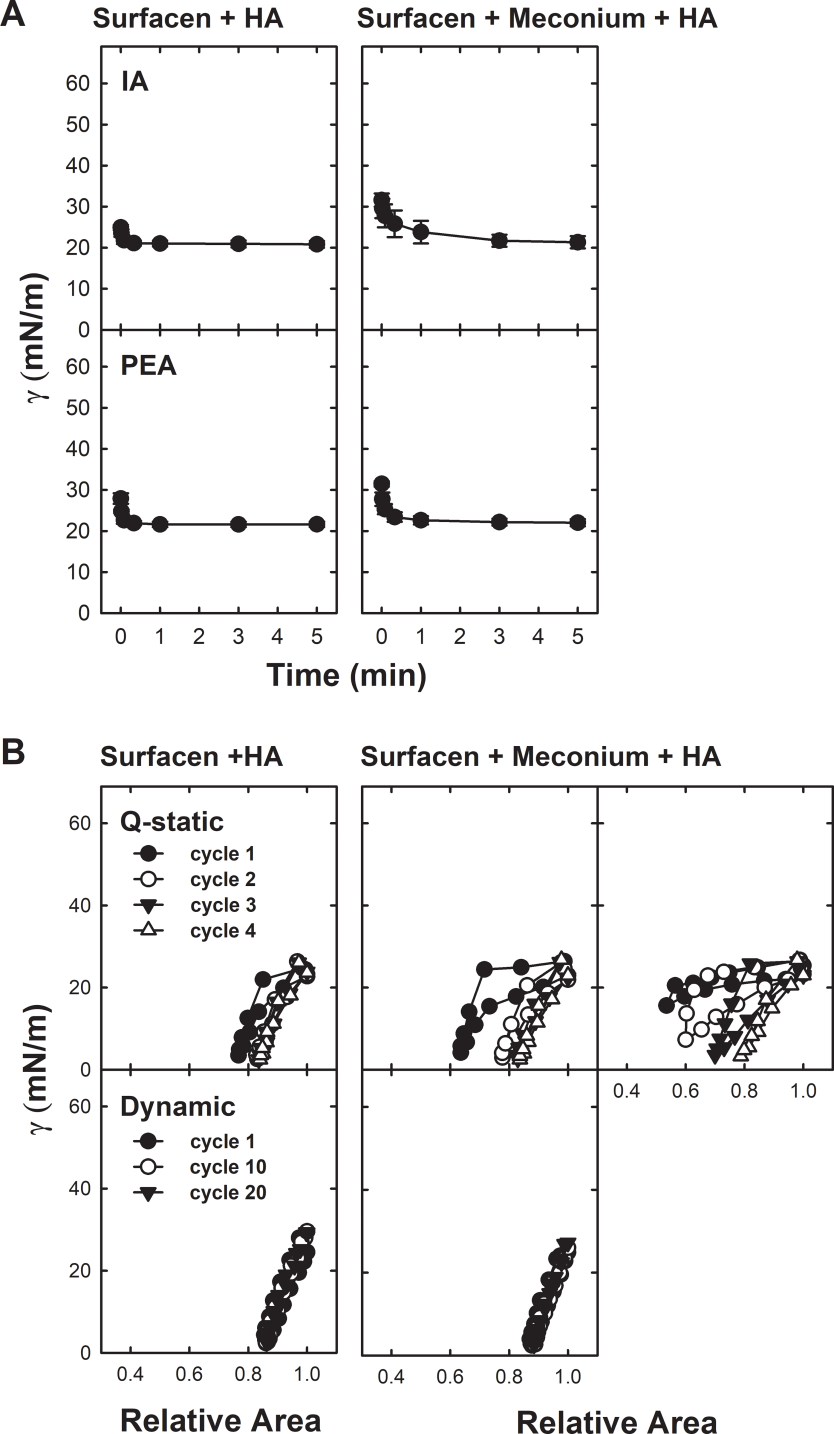
Effect of meconium on the surface activity of Surfacen as evaluated by the Captive Bubble Surfactometer. A) Initial and Post expansion adsorption of Surfacen in the absence (left) and in the presence (right) of meconium. B) Quasi- static (Q-static, upper panels) and dynamic (lower panels) compression-expansion isotherms of Surfacen films formed in the absence (left) or in the presence (right) of meconium.

Fig 2 compares interfacial adsorption kinetics of Surfacen and Surfacen pre-exposed to meconium, as assessed after deposition at the interface of the air bubble in the captive bubble surfactometer. Initial and post-expansion adsorption (Fig 2A) as well as quasi-static and dynamic cycling isotherms (Fig 2B) of Surfacen can be compared with and without pre-incubation with meconium. Clean Surfacen adsorbs to form a stable surface film with a minimum equilibrium surface tension of 23±0.5 mN/m within the first seconds after application. Surfacen exposed to meconium adsorbed as fast as clean Surfacen, although it reached a somehow higher surface tension (33.5±9.4 mN/m after five minutes). Re-adsorption of excess material upon expansion of the bubble showed similar results (Fig 2 A). Variability of the isotherms obtained from meconium-treated samples is typically very high, independently of the number of experiments and samples tested, probably as a consequence of the particulated and heterogeneous nature of meconium itself.

Cycling isotherms obtained from clean Surfacen films show practically no compression/expansion hysteresis along the successive quasi-static or dynamic cycles (Fig 2B). Surfacen reaches a minimum surface tension of 2.4±0.15 mN/m with only 15% compression during dynamic cycles. In contrast, the isotherms of Surfacen exposed to meconium exhibited again high variability of the cycling isotherms. In Fig 2B three representative experiments (from a total of nine repetitions) of such variable behavior are summarized, under quasi-static as well as dynamic cycles, while the experiments were carried out under identical conditions. Under quasi-static compression/expansion cycling, hysteresis was still limited but much larger area compression was needed in some experiments in order to get values of minimal surface tension of 19.6 mN/m. However, no effect of meconium was observed in other experiments where pre-exposure to meconium still maintained ability to get minimal surface tension, similar (i.e. at the third and fourth cycles) to clean Surfacen. In contrast, meconium-exposed Surfacen always needed larger area compression and exhibited marked hysteresis in both quasi-static and dynamic cycling.

**Fig 3:**
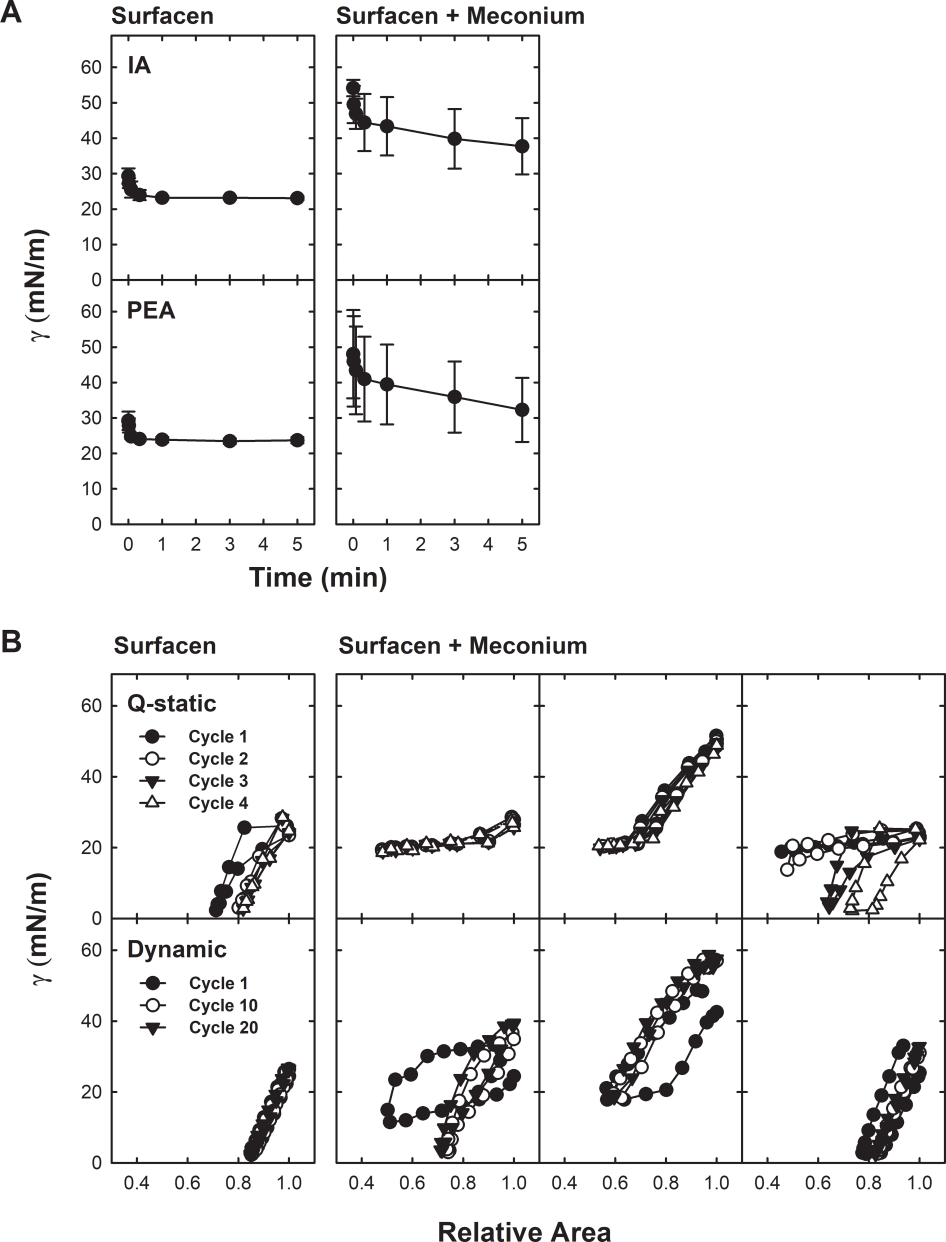
Reversion by HA of the inhibition of Surfacen by meconium. A) Initial and Post expansion adsorption of Surfacen plus HA in the absence (left) or in the presence (right) of meconium. B) Quasi- static (Q-static, upper panels) and dynamic (lower panels) compression-expansion isotherms of Surfacen plus HA films formed in the absence (left panels) or in the presence (right panels) of meconium.

On the other hand, isotherms obtained from films formed by the mixture Surfacen/HA (Fig 3) always exhibited a similar behavior independently of being or not pre-exposed to meconium, with meconium-exposed Surfacen still showing practically no compression/expansion hysteresis and reaching a minimal tension of 2.8±0.6 mN/m with only 20% of area compression. Only the first quasi-static cycle exhibited a marginal hysteresis, which was lost in the subsequent cycles, probably as a consequence of an equilibration/reorganization of the film during compression. In summary, addition of HA totally restored the behavior of Surfacen in the presence of meconium during Q-static and dynamic compression-expansion cycling, which finally produced very low surface tension. Furthermore, HA-treated Surfacen showed a very consistent interfacial behavior, with no variability between experiments with or without meconium pre-exposure.

**Fig 4:**
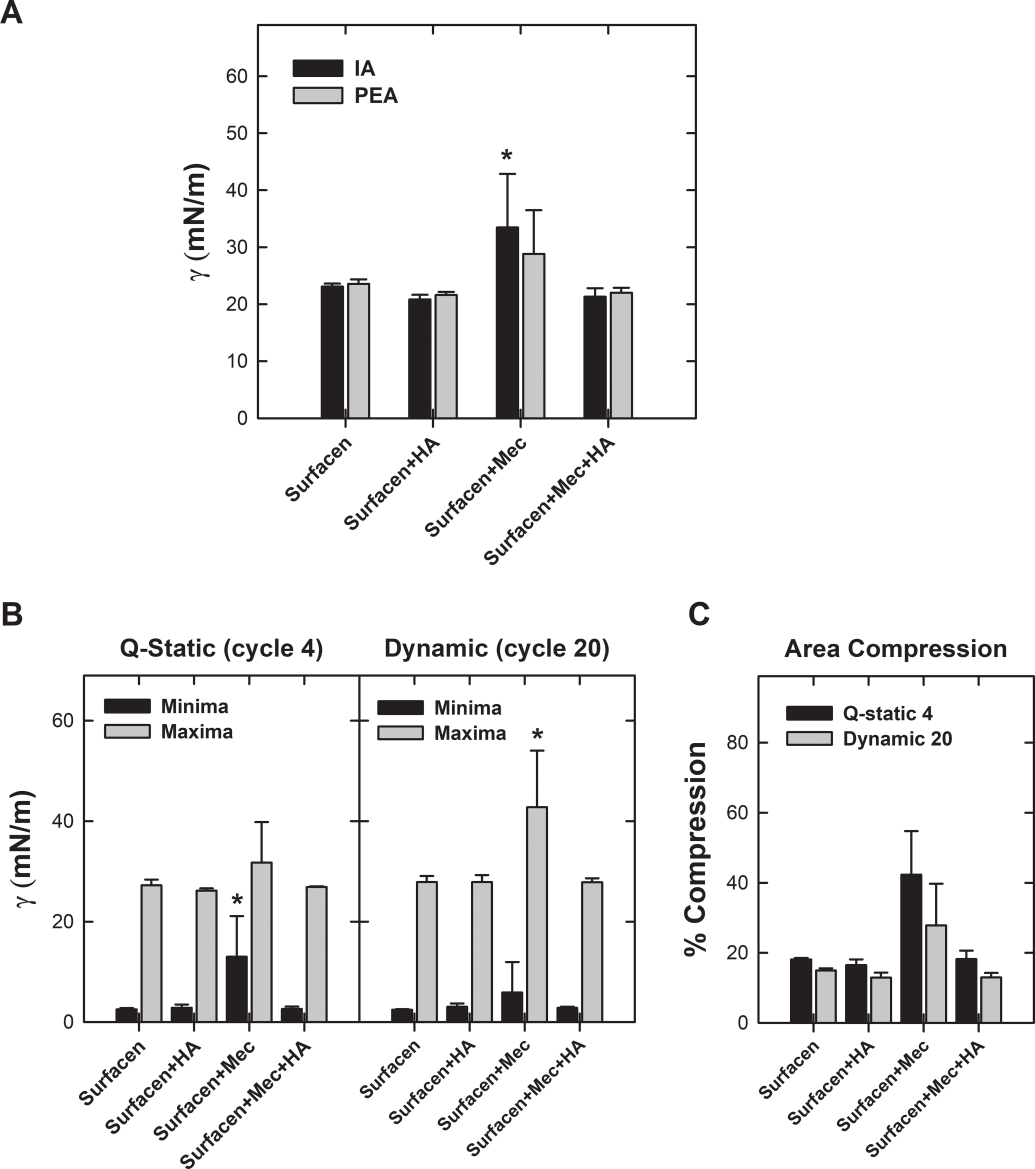
Parameters defining the surface behavior of Surfacen in the absence and presence of meconium and/or HA. A) Minimum surface tension after Initial or Post expansion adsorption of Surfacen alone or in the presence of meconium and/or HA. B) Minimum and maximum surface tension after Quasi-static (fourth cycle) or dynamic (tenth cycle) compression-expansion isotherms of films formed by Surfacen alone or Surfacen pre-mixed with meconium and/or HA. C) Percentage of area reduction required to get minimal tension by films formed by Surfacen alone or Surfacen in the presence of meconium and/or HA. Asterisk indicates that the mean is statistically different to the compared values (p<0.05) (see text).

Fig 4 summarizes the most relevant parameters defining the behavior of Surfacen challenged with meconium at the CBS and the reversion by HA. Panel A illustrates the equilibrium surface tension reached upon adsorption, either initially or after bubble expansion, of meconium- and/or HA-pretreated Surfacen. Equilibrium surface tension reached during initial adsorption by HA pre-exposed surfactant was not statistically different from that produced by films made of Surfacen alone. Surfacen pre-mixed with meconium lead to significantly higher average surface tension (p = 0.005) than clean Surfacen or Surfacen pre-mixed with meconium and HA. Differences in minimal tension during post-expansion adsorption were not found to be statistically significant, due to the large standard deviation as a consequence of the high heterogeneity in the behavior of replicas, probably associated with the intrinsic complexity and heterogeneity of meconium as indicated above. Panel B in Fig 4 illustrates minimum and maximum surface tension at the end of the 4th quasi-static or of the 20th dynamic compression-expansion cycles of meconium- and/or HA-pretreated Surfacen. Surfacen pre-exposed to meconium in the absence of HA produced significantly higher surface tension (p = 0.006) than clean Surfacen or Surfacen exposed to meconium in the presence of HA, during q-static cycling. The minimal tension was not statistically different during dynamic cycling, indicating that the films could be at least partly depurated of deleterious components during rapid compression. However, meconium-exposed Surfacen produced significantly higher maximal tensions (p = 0.005) at the end of the expansion part of the dynamic isotherms than clean Surfacen or Surfacen that had been exposed to meconium in the presence of HA. This indicates that although rapid cycling could aid to continuously depurate the less surface-active components from meconium-contaminated interfacial films, adsorption is still defective to properly replenish the films with new arriving components during the expansion periods. Optimal behavior during both q-static or dynamic cycling was fully preserved in the presence of HA. Panel C summarizes the area reduction needed to reach minimum surface tension at the end of the 4th quasi-static or 20th dynamic compression-expansion cycles. Surfacen exposed to meconium needs a reduction in area of almost 40% to get similar surface tension to that reached by Surfacen alone or Surfacen plus HA at the 4th quasi-static cycle (see Figs 2 and 3).

**Fig 5:**
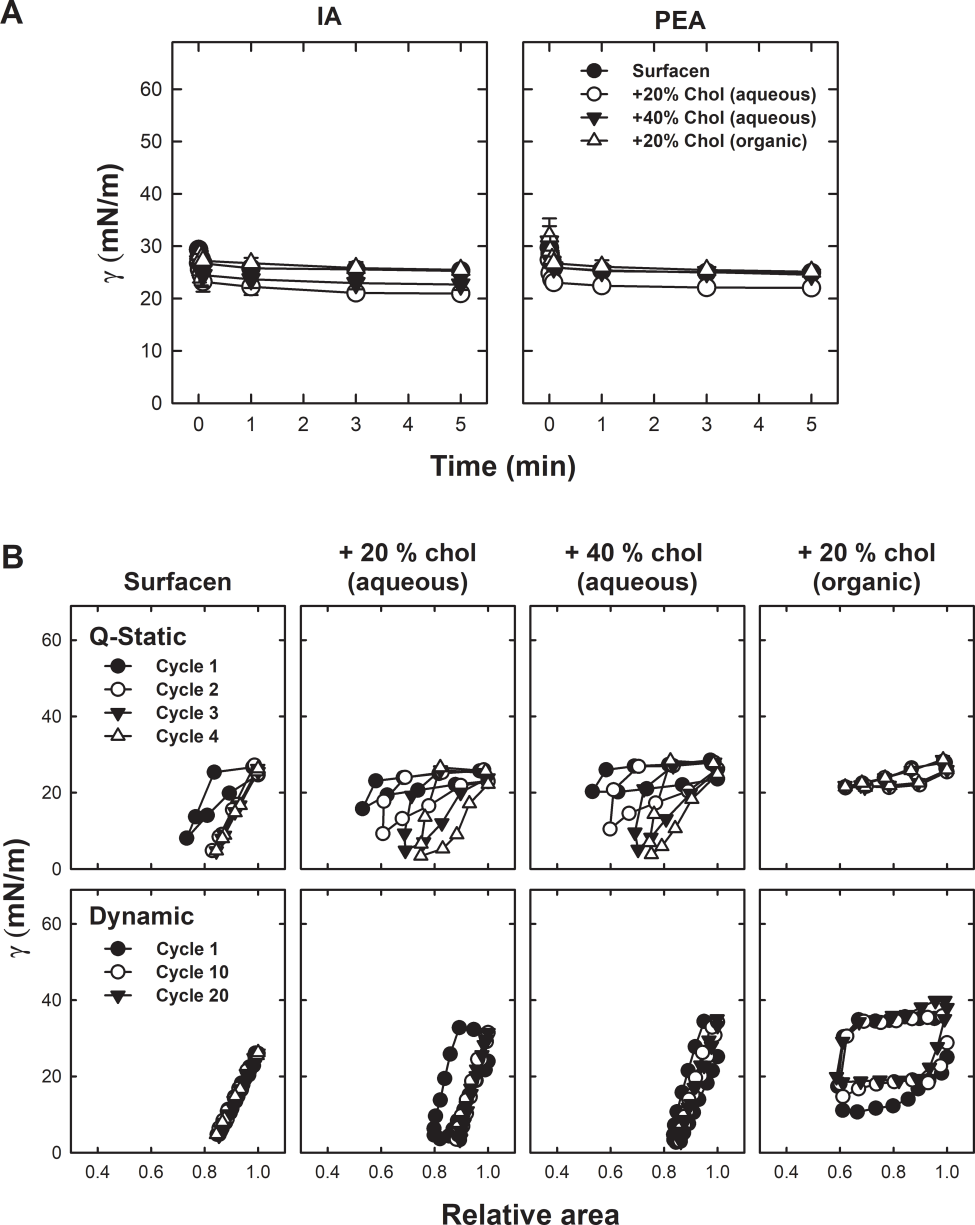
Surface behavior of Surfacen incorporating aqueous or organic solvent cholesterol. Surfacen has been either exposed to MβCD-cholesterol (aqueous) or extracted in organic solvent and mixed with cholesterol before reconstituting the mixture again as aqueous suspension. A. Interfacial initial and post-expansion adsorption (IA and PEA) of Surfacen plus 20% or 40% (with respect to phospholipids) of MβCD-cholesterol (aqueous) or cholesterol (organic), as assessed in the CBS at 37°C. B. Representative compression-expansion isotherms obtained during Q-static or dynamic cycling. Data are means ± standard deviation in IA and PEA plots. A representative experiment is shown in each panel of cycling isotherms, after several repetitions (n>3) from each sample.

Considering the high variability of the effect of meconium on Surfacen and the fact that the inactivation of surfactant by meconium has been proven to rely on the amount of cholesterol transferred from meconium [27], we decided to compare meconium inactivation with the effect of the incorporation of an excess of cholesterol into Surfacen complexes. Fig 5A shows that the addition of 20% (w/w) cholesterol to Surfacen produced different effects on its biophysical behavior depending on the strategy used to expose Surface to the sterol. When 20% (w/w) cholesterol was incorporated into Surfacen mixtures that were previously extracted by organic solvents and later reconstituted again as cholesterol-containing aqueous suspensions, it produced a clearly deleterious effect on the Q-static and dynamic compression-expansion isotherms, which required an area reduction of around 40% to reach minimal surface tensions of only 20 mN/m. In contrast, exposure of Surfacen to 20% (w/w) cholesterol solubilized by cyclodextrin had a very limited inhibitory effect on its biophysical behavior. In this case, Q-static cycles required larger area reduction (around 50% in the first cycle) at first instance, but in the fourth cycle compressibility was restored to the same values as in cholesterol-free Surfacen. However, all compression-expansion cycles showed larger hysteresis in comparison to those in the isotherms of cholesterol-free Surfacen. In the dynamic compression-expansion isotherms, only the first cycle had a larger hysteresis. Cholesterol (added as organic mixture or solubilized by cyclodextrin) had not apparent effect on either initial or post-expansion adsorption, independently of the incorporation method.

**Fig 6:**
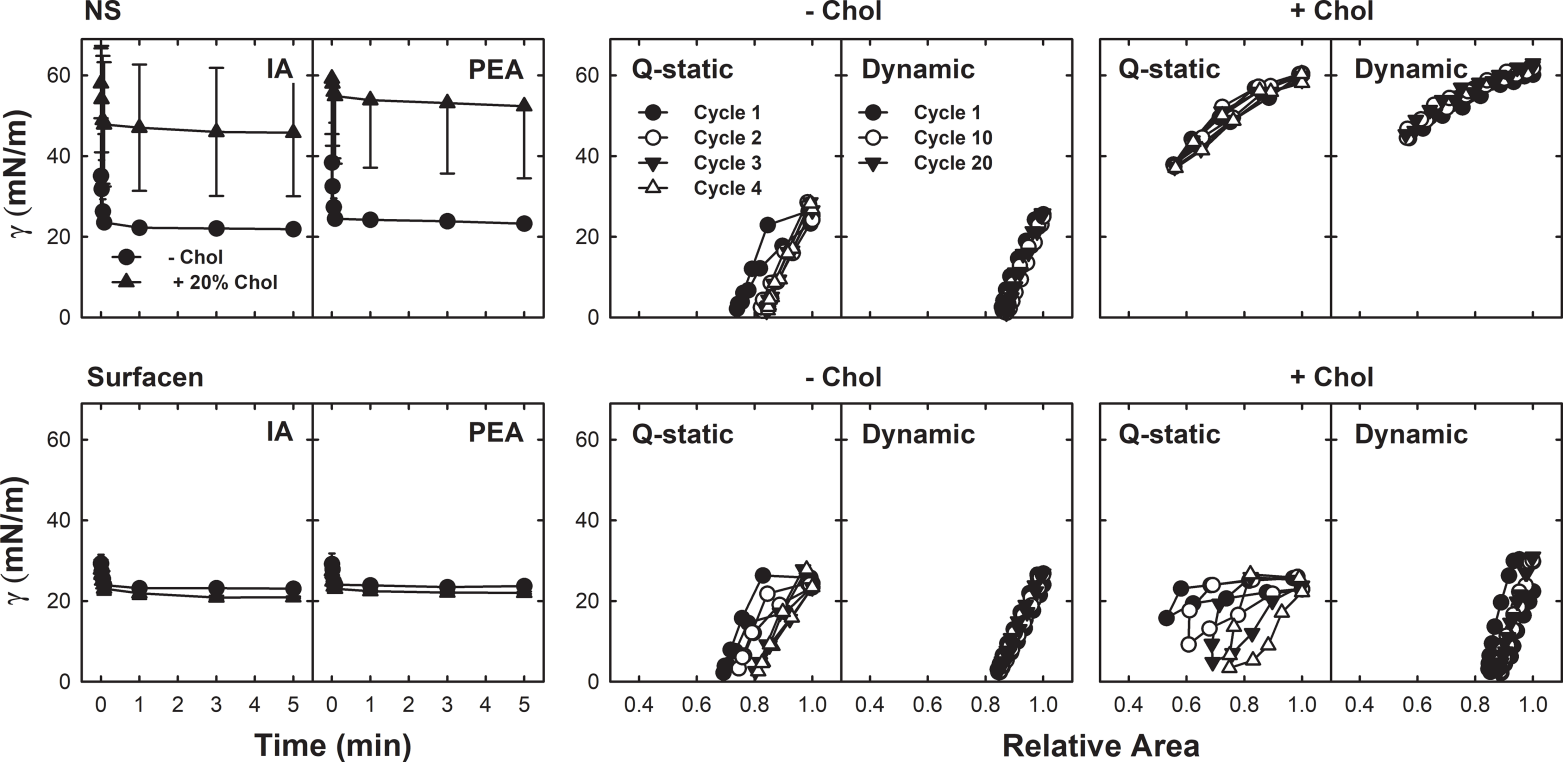
Surface behavior of Surfacen or porcine native surfactant exposed to 20% MβCD-cholesterol as assessed in the CBS at 37°C. Data are means ± standard deviation in IA and PEA plots. A representative experiment is shown in each panel of compression-expansion isotherms, after several repetitions (n = 4) from each sample.

The inhibitory effect of the incorporation of cholesterol to Surfacen in organic solvent mixtures seems to be associated to an effective increase in the proportion of cholesterol up to 16% with respect to phospholipids (Table 1) once the Surfacen/cholesterol mixtures were again reconstituted as aqueous solutions. In contrast, exposure of Surfacen to cholesterol-loaded MβCD only increased the proportion of the sterol into the surfactant complexes up to 11%. It has been determined that the physiological level of cholesterol in endogenous native lung surfactant can be around 5–10% with respect to phospholipid mass. As Surfacen production ends in an almost complete depletion of cholesterol [35], the ulterior incorporation of proportions of cholesterol that do not overpass by as much as 10% could still maintain physiological limits with no apparent negative effects in Surfacen biophysical behavior. We therefore proceeded to test the effect of the exposure of Surfacen to much higher levels of cholesterol, which could increase the actual incorporation of cholesterol into the complexes of this clinical surfactant, mimicking certain pathological contexts. To this purpose, we incubated Surfacen with 40% (w/w) cholesterol solubilized in MβCD, as this could mimic situations in which high amounts of cholesterol could be mobilized towards surfactant, such as in cases of meconium aspiration. Fig 5 illustrates that the behavior of Surfacen exposed to 40% cholesterol solubilized by cyclodextrin was not too different from the behavior of Surfacen exposed to 20% solubilized cholesterol. Table 1 shows that there are important differences in the extent of incorporation of cholesterol into Surfacen complexes compared to the incorporation into native lung surfactant upon exposure to comparable amounts of solubilized cholesterol. Surfacen seems always to incorporate less cholesterol in comparison with native surfactant membranes. When cholesterol is added to surfactant mixtures in organic solvent, both surfactant phospholipids and cholesterol are fully mixed and incorporated into common reconstituted membranes. When cholesterol is solubilized by aqueous mobilizing agents, such as bile acids or cyclodextrin, and added directly to the aqueous suspensions of surfactants, the incorporation of cholesterol could depend on factors such as the composition and structure of the membranes. In this sense, it seems that the susceptibility of each surfactant to incorporate solubilized cholesterol could differ substantially, with a possible threshold above which further addition of soluble cholesterol to the medium does not increase the content of cholesterol any further in surfactant complexes (Table 1). Fig 6 illustrates the different biophysical behavior of native surfactant complexes and of the clinical surfactant Surfacen once exposed to water-soluble cholesterol. Native surfactant membranes were more susceptible to incorporation of cholesterol than the clinical surfactant (Table 1) and as a consequence, native surfactant exposed to solubilized cholesterol exhibits a worse biophysical behavior than cholesterol-exposed Surfacen (Fig 6) at the conditions imposed by our CBS tests, designed to mimic physiologically-meaningful constraints.

**Table 1:** Cholesterol incorporation into Surfacen or native surfactant (NS) membranes and its effect on interfacial behavior as assayed in CBS.

	% cholesterol				
Added Chol	0	20% MβCD	40% MβCD	20% organic	40% organic
**NS**	5	31 (+)			
**Surfacen**	n.d.	11.2 (**-**)	8 (**-**)	16.4 (+)	17 (+)

Numbers indicate percentage of cholesterol by mass with respect to phospholipid upon determination as indicated in Materials and Methods. n.d.: not detected.

(+) Surface activity inhibited, as determined at the CBS

(-) Absence of inhibition

## Discussion

This study has analyzed the sensitivity of Surfacen, a clinical surfactant currently in use, to inhibition by pathogenic substances such as serum (leaked into alveolar spaces in cases of lung injury, inflammation and edema) or meconium (aspirated by babies suffering MAS). The inhibition has been tested *in vitro*, under conditions that have been recently optimized [19] to mimic truly pathogenic contexts, including high concentrations of surfactant (20 mg/mL) and serum (100 mg/mL protein). Concentration of surfactant used for inhibition studies is a crucial factor, because a critical concentration is required for a clinical surfactant to reorganize from a dispersed suspension of membranous complexes into large surface-associated membrane networks, depending on the particular surfactant and ionic conditions [25, 36].

Previously, only a few studies have compared different surfactant preparations against inactivation by plasma proteins, and it was found that different surfactants have differential sensitivity to inhibition [37]. The susceptibility of Surfacen to inactivation had not been previously tested, in spite of Surfacen being extensively applied in Cuban hospitals to treat babies suffering or at risk of NRDS. Previous results have also shown the potential of using polymers to reduce surfactant inactivation [12, 16, 20]. Anionic polymers such as HA or chitosan have more advantages than non-ionic ones, as they require lower concentrations to revert inhibition and therefore impose a lower viscosity to the final surfactant-polymer mixture [9]. The exposure to HA showed similar effects towards different surfactant preparations, indicating that they are all reactivated by a common mechanism. We recently reported that polymers not only enhance interfacial adsorption but also restore compressibility of surfactant films during compression-expansion cycling in the presence of inhibitors [19]. It was therefore concluded that the reversion mechanism is somehow general for surfactants and not specific for the inhibitory agents tested. Further studies concluded that exposure to polymers above concentration thresholds at which polymer chains entangle causes substantial changes in surfactant membrane structure [38]. These changes include extensive packing, dehydration and structural depuration ending in a highly active structure able to overpass inhibition by serum or meconium. Many of these changes were irreversible, suggesting that pre-exposure to polymers such as HA could be introduced at the production protocols of clinical surfactants as a mean to deliver much better therapeutic preparations.

The results of the current study illustrate the inhibitory effect of serum or meconium towards one more therapeutic surfactant, Surfacen, and extend the ability of HA to impart a substantial resistance to inactivation to this extensively used clinical preparation. Mixtures of Surfacen with HA challenged by different inhibitory agents resulted in restored activity, comparable to that of pristine Surfacen. HA did not interfere with surface activity of Surfacen itself, suggesting that addition or treatment of Surfacen with polymers such as HA could enhance its activity under the challenging conditions imposed by pathologies such as meconium aspiration syndrome (MAS) in neonates or acute respiratory distress syndrome (ARDS) in children and adults.

Cholesterol is the major neutral lipid in pulmonary surfactant where it can be encountered at a concentration of up to 10% by mass (20 mol %) with respect to phospholipids. The presence of cholesterol in surfactant has long been known; however, the role of cholesterol in surfactant remains uncertain [23]. On the other hand, presence of an excess of cholesterol has been linked with impairment of surfactant function associated with severe pathologies such as ARDS [39, 40]. Traditionally, cholesterol inhibition has been studied at the bench by adding cholesterol dissolved in organic solvent into organic extracted surfactant mixtures. However, incorporation into surfactant complexes of cholesterol from organic solvent mixtures is not physiologically relevant, as cholesterol is made available in vivo as part of structures that solubilize it in water, such as lipoproteins or bile salt micelles. A variety of methods have been used to model mobilization of cholesterol from and into lipid membranes with variable success. We have applied here an incorporation method based on the exposure of surfactant complexes to cholesterol-loaded cyclodextrin. Previous studies have shown that cholesterol molecules are easily incorporated from cholesterol/cyclodextrin complexes into cell membranes [41] or into surfactant complexes [27], the incorporation being well reproducible and needing short incubation times. It has been reported that at least 20% of cholesterol (w/w, cholesterol/phospholipid) is required to observe surfactant dysfunction [9, 22, 24, 40]. We have therefore evaluated the incorporation of 20% of cholesterol but in its solubilized aqueous form. Interestingly, we found that not all the cholesterol was actually incorporated into the Surfacen membranes (see Table 1), and as a consequence, its biophysical behavior was not dramatically affected because the final proportion of cholesterol did not overpass physiological-like levels by much. We compared this behavior with that of native surfactant, which could somehow reproduce *in vitro* conditions thought to occur during meconium aspiration in vivo events, where cholesterol amounts in bronchoalveolar lavages become very high [42]. The differences between membranes from native versus the clinical surfactant with respect to their capacity to incorporate cholesterol are remarkable. It seems that a cholesterol-containing surfactant, such as the native preparation obtained from porcine lungs, could be somehow ‘primed’ to accept more sterol delivered from soluble structures such as the cyclodextrin complexes. Gunasekara et al [22] confirmed the hypothesis predicted by Discher et al [43] that cholesterol could associate with DPPC-enriched membrane regions rather than with the more fluid matrix, by using the exogenous surfactant preparation BLES. Previous studies by our group showed that condensed domains in Surfacen membranes, presumably DPPC-enriched, were larger and occupied a larger fractional surface than condensed domains in native lung surfactant or its organic extract as seen by epifluorescence microscopy [35]. Our current results suggest that a higher proportion of condensed membrane domains, as exhibited by Surfacen, is not at all associated with a higher cholesterol incorporation, but the opposite. We therefore propose that the transference of cholesterol from soluble structures into surfactant membranes could start at the most fluid regions, independently of where cholesterol finally partitions as a consequence of ulterior lateral diffusion and equilibration. The consequence is that Surfacen could be less prone to accepting solubilized cholesterol than the native surfactant complexes or other clinical preparations with less proportion of condensed membranes.

A main compositional difference shared by Surfacen and other therapeutic preparations such as Alveofact, Infasurf or Survanta with respect to native surfactant is the significant reduction in cholesterol content [29, 44, 45]. It has been argued that a low-cholesterol content could permit exogenous surfactants to better resist exposure to cholesterol-rich pathogenic contexts before reaching the inhibitory thresholds of cholesterol in surfactant. Our results suggest that incorporation of cholesterol into surfactant might not be a mere question of adding up the exogenous cholesterol to the endogenous proportion. Exposure of Surfacen to higher amounts of cholesterol did not imply in fact a proportional increase in cholesterol incorporation and confirms that a maximum cholesterol stoichiometry seems to be acceptable in this complex [22]. The consequence is that Surfacen does not seem to reach apparently the proportions of cholesterol that could compromise the compressibility and mechanical stability of its interfacial films. This is likely a consequence of the particular lipid composition of Surfacen and its concomitant membrane phase structure, although other differences between Surfacen and native surfactant complexes, such as the level of complexity of the membrane networks or the lack of proteins such as SP-A, could also contribute to the different responses to cholesterol incorporation in a manner difficult to predict. In principle, one would expect that more complex three-dimensional structures such as those in native surfactant, as they are assembled and secreted in vivo, should be more protected from exposure to soluble cholesterol transporters and therefore be less susceptible to cholesterol transfer. This is clearly not the case. Presence of protein SP-A has been also proposed as protective of surfactant inhibition, again as a consequence of the protein promoting the assembly of more compact and complex lipid/protein membrane networks [46–48]. Our results seem to indicate that these differences in membrane complexity potentially promoted by SP-A are not associated with reduced cholesterol incorporation but with transfer of larger amounts of sterol and higher susceptibility to functional inhibition. The different physico-chemical properties in terms of lipid packing, fluidity, or viscosity of the cholesterol-free membranes of Surfacen, and possibly of other cholesterol-depleted surfactants, could impose an intrinsic barrier to cholesterol import by cholesterol-mobilizing agents and therefore provide a genuine resistance to cholesterol inhibition. In this sense, Surfacen could constitute an excellent clinical preparation for treating cases where cholesterol inactivation is expected as an important pathogenic factor, as it occurs in patients of MAS. To what extent the resistance to cholesterol inhibition is a general feature of any cholesterol-free clinical preparation or whether it is a combination of the lack of cholesterol and a particular composition/structure generated as a consequence of the procedures applied during surfactant production is something that requires further investigation. It remains also as an open question to what extent this resistance to incorporate exogenous cholesterol, and therefore to resist certain inhibitory contexts, would be also exhibited under true *in vivo* challenging conditions. Optimizing a composition/structure with minimal cholesterol incorporation capabilities could contribute to implementing novel strategies in the production of the exogenous surfactants that are still required to treat the most challenging respiratory pathologies.

## References

[1] Perez-Gil J. Structure of pulmonary surfactant membranes and films: the role of proteins and lipid-protein interactions. Biochim Biophys Acta. 2008;1778(7–8):1676–95. 10.1016/j.bbamem.2008.05.003 18515069

[2] Sardesai S, Biniwale M, Wertheimer F, Garingo A, Ramanathan R. Evolution of surfactant therapy for respiratory distress syndrome: past, present, and future. Pediatr Res. 2017;81(1–2):240–8. 10.1038/pr.2016.203 27706130

[3] Taeusch HW. Treatment of acute (Adult) respiratory distress syndrome. The holy grail of surfactant therapy. Biol Neonate. 2000;77 Suppl 1:2–8. 10828579 10.1159/000047050

[4] Dushianthan A, Grocott MP, Postle AD, Cusack R. Acute respiratory distress syndrome and acute lung injury. Postgrad Med J. 2011;87(1031):612–22. 10.1136/pgmj.2011.118398 21642654

[5] Park KH, Bae CW, Chung SJ. In vitro effect of meconium on the physical surface properties and morphology of exogenous pulmonary surfactant. J Korean Med Sci. 1996;11(5):429–36. 10.3346/jkms.1996.11.5.429 8934399 PMC3054187

[6] Rubenfeld GD, Caldwell E, Peabody E, Weaver J, Martin DP, Neff M, et al Incidence and outcomes of acute lung injury. N Engl J Med. 2005;353(16):1685–93. 10.1056/NEJMoa050333 16236739

[7] Matthay MA, Zemans RL. The acute respiratory distress syndrome: pathogenesis and treatment. Annu Rev Pathol. 2011;6:147–63. 10.1146/annurev-pathol-011110-130158 20936936 PMC3108259

[8] Wiedemann JR, Saugstad AM, Barnes-Powell L, Duran K. Meconium aspiration syndrome. Neonatal Netw. 2008;27(2):81–7. 10.1891/0730-0832.27.2.81 18431962

[9] Zuo YY, Veldhuizen RA, Neumann AW, Petersen NO, Possmayer F. Current perspectives in pulmonary surfactant—inhibition, enhancement and evaluation. Biochim Biophys Acta. 2008;1778(10):1947–77. 10.1016/j.bbamem.2008.03.021 18433715

[10] Lopez-Rodriguez E, Perez-Gil J. Structure-function relationships in pulmonary surfactant membranes: from biophysics to therapy. Biochim Biophys Acta. 2014;1838(6):1568–85. 10.1016/j.bbamem.2014.01.028 24525076

[11] Calkovska A, Some M, Linderholm B, Johansson J, Curstedt T, Robertson B. Biophysical and physiological properties of porcine surfactant enriched with polymyxin B. Biol Neonate. 2005;88(2):101–8. 10.1159/000085524 15860913

[12] Calkovska A, Some M, Linderholm B, Curstedt T, Robertson B. Therapeutic effects of exogenous surfactant enriched with dextran in newborn rabbits with respiratory failure induced by airway instillation of albumin. Pulm Pharmacol Ther. 2008;21(2):393–400. 10.1016/j.pupt.2007.10.003 18032077

[13] Calkovska A, Mokra D, Drgova A, Zila I, Javorka K. Bronchoalveolar lavage with pulmonary surfactant/dextran mixture improves meconium clearance and lung functions in experimental meconium aspiration syndrome. Eur J Pediatr. 2008;167(8):851–7. 10.1007/s00431-007-0596-7 17952467

[14] Lu KW, Goerke J, Clements JA, Taeusch HW. Hyaluronan reduces surfactant inhibition and improves rat lung function after meconium injury. Pediatr Res. 2005;58(2):206–10. 10.1203/01.PDR.0000169981.06266.3E 16055934

[15] Lu KW, Robertson B, Taeusch HW. Dextran or polyethylene glycol added to curosurf for treatment of meconium lung injury in rats. Biol Neonate. 2005;88(1):46–53. 10.1159/000084458 15767742

[16] Lu KW, Goerke J, Clements JA, Taeusch HW. Hyaluronan decreases surfactant inactivation in vitro. Pediatr Res. 2005;57(2):237–41. 10.1203/01.PDR.0000150726.75308.22 15585679

[17] Herting E, Rauprich P, Stichtenoth G, Walter G, Johansson J, Robertson B. Resistance of different surfactant preparations to inactivation by meconium. Pediatr Res. 2001;50(1):44–9. 10.1203/00006450-200107000-00010 11420417

[18] Lu KW, Perez-Gil J, Taeusch H. Kinematic viscosity of therapeutic pulmonary surfactants with added polymers. Biochim Biophys Acta. 2009;1788(3):632–7. 10.1016/j.bbamem.2009.01.005 19366601 PMC2671574

[19] Lopez-Rodriguez E, Ospina OL, Echaide M, Taeusch HW, Perez-Gil J. Exposure to polymers reverses inhibition of pulmonary surfactant by serum, meconium, or cholesterol in the captive bubble surfactometer. Biophys J. 2012;103(7):1451–9. 10.1016/j.bpj.2012.08.024 23062337 PMC3471484

[20] Lu JJ, Cheung WW, Yu LM, Policova Z, Li D, Hair ML, et al The effect of dextran to restore the activity of pulmonary surfactant inhibited by albumin. Respir Physiol Neurobiol. 2002;130(2):169–79. 12380007 10.1016/s0034-5687(02)00006-3

[21] Lu KW, Perez-Gil J, Echaide M, Taeusch HW. Pulmonary surfactant proteins and polymer combinations reduce surfactant inhibition by serum. Biochim Biophys Acta. 2011;1808(10):2366–73. 10.1016/j.bbamem.2011.06.013 21741354 PMC3156878

[22] Gunasekara L, Schurch S, Schoel WM, Nag K, Leonenko Z, Haufs M, et al Pulmonary surfactant function is abolished by an elevated proportion of cholesterol. Biochim Biophys Acta. 2005;1737(1):27–35. 10.1016/j.bbalip.2005.09.002 16216549

[23] Keating E, Rahman L, Francis J, Petersen A, Possmayer F, Veldhuizen R, et al Effect of cholesterol on the biophysical and physiological properties of a clinical pulmonary surfactant. Biophys J. 2007;93(4):1391–401. 10.1529/biophysj.106.099762 17526587 PMC1929052

[24] Leonenko Z, Gill S, Baoukina S, Monticelli L, Doehner J, Gunasekara L, et al An elevated level of cholesterol impairs self-assembly of pulmonary surfactant into a functional film. Biophys J. 2007;93(2):674–83. 10.1529/biophysj.107.106310 17483162 PMC1896251

[25] Gunasekara L, Schoel WM, Schurch S, Amrein MW. A comparative study of mechanisms of surfactant inhibition. Biochim Biophys Acta. 2008;1778(2):433–44. 10.1016/j.bbamem.2007.10.027 18036553

[26] Bernardino de la Serna J, Perez-Gil J, Simonsen AC, Bagatolli LA. Cholesterol rules: direct observation of the coexistence of two fluid phases in native pulmonary surfactant membranes at physiological temperatures. J Biol Chem. 2004;279(39):40715–22. 10.1074/jbc.M404648200 15231828

[27] Lopez-Rodriguez E, Echaide M, Cruz A, Taeusch HW, Perez-Gil J. Meconium impairs pulmonary surfactant by a combined action of cholesterol and bile acids. Biophys J. 2011;100(3):646–55. 10.1016/j.bpj.2010.12.3715 21281579 PMC3030210

[28] Moreno OV, López ML, Dieppa FD, López MAP, Abad AA, Rivero GJ, et al Estudio de la eficacia del SURFACEN en el Distres Respiratorio del recién nacido. Rev Cubana Pediatria. 1999;71((2)):60–71.

[29] Blanco O, Perez-Gil J. Biochemical and pharmacological differences between preparations of exogenous natural surfactant used to treat Respiratory Distress Syndrome: role of the different components in an efficient pulmonary surfactant. Eur J Pharmacol. 2007;568(1–3):1–15. 10.1016/j.ejphar.2007.04.035 17543939

[30] Manzanares D, Diaz E, Alfonso W, Escobar A, Colome H, Muñoz MC, et al Surfactante pulmonar porcino. República de Cuba 1995;A 61:35–42 K.

[31] Taeusch HW, Bernardino de la Serna J, Perez-Gil J, Alonso C, Zasadzinski JA. Inactivation of pulmonary surfactant due to serum-inhibited adsorption and reversal by hydrophilic polymers: experimental. Biophys J. 2005;89(3):1769–79. 10.1529/biophysj.105.062620 15923228 PMC1366680

[32] Schurch S, Green FH, Bachofen H. Formation and structure of surface films: captive bubble surfactometry. Biochim Biophys Acta. 1998;1408(2–3):180–202. 9813315 10.1016/s0925-4439(98)00067-2

[33] Rouser G, Siakotos AN, Fleischer S. Quantitative analysis of phospholipids by thin-layer chromatography and phosphorus analysis of spots. Lipids. 1966;1(1):85–6. 10.1007/BF02668129 17805690

[34] DiRienzo JA, Casanoves F, Balzarini MG, Gonzalez L, Tablada M, Robledo CW. InfoStat versión 2018 Grupo InfoStat, FCA. 2018;Universidad Nacional de Córdoba, Argentina.

[35] Blanco O, Cruz A, Ospina OL, Lopez-Rodriguez E, Vazquez L, Perez-Gil J. Interfacial behavior and structural properties of a clinical lung surfactant from porcine source. Biochim Biophys Acta. 2012;1818(11):2756–66. 10.1016/j.bbamem.2012.06.023 22771553

[36] Holm BA, Wang Z, Notter RH. Multiple mechanisms of lung surfactant inhibition. Pediatr Res. 1999;46(1):85–93. 10400140 10.1203/00006450-199907000-00015

[37] Seeger W, Grube C, Gunther A, Schmidt R. Surfactant inhibition by plasma proteins: differential sensitivity of various surfactant preparations. Eur Respir J. 1993;6(7):971–7. 8370446

[38] Lopez-Rodriguez E, Cruz A, Richter RP, Taeusch HW, Perez-Gil J. Transient exposure of pulmonary surfactant to hyaluronan promotes structural and compositional transformations into a highly active state. J Biol Chem. 2013;288(41):29872–81. 10.1074/jbc.M113.493957 23983120 PMC3795285

[39] Markart P, Ruppert C, Wygrecka M, Colaris T, Dahal B, Walmrath D, et al Patients with ARDS show improvement but not normalisation of alveolar surface activity with surfactant treatment: putative role of neutral lipids. Thorax. 2007;62(7):588–94. 10.1136/thx.2006.062398 17287298 PMC2117258

[40] Vockeroth D, Gunasekara L, Amrein M, Possmayer F, Lewis JF, Veldhuizen RA. Role of cholesterol in the biophysical dysfunction of surfactant in ventilator-induced lung injury. Am J Physiol Lung Cell Mol Physiol. 2010;298(1):L117–25. 10.1152/ajplung.00218.2009 19897745

[41] Hartel S, Diehl HA, Ojeda F. Methyl-beta-cyclodextrins and liposomes as water-soluble carriers for cholesterol incorporation into membranes and its evaluation by a microenzymatic fluorescence assay and membrane fluidity-sensitive dyes. Anal Biochem. 1998;258(2):277–84. 9570841 10.1006/abio.1998.2594

[42] Autilio C, Echaide M, De Luca D, Perez-Gil J. Controlled hypothermia may improve surfactant function in asphyxiated neonates with or without meconium aspiration syndrome. PLoS One. 2018;13(2):e0192295 10.1371/journal.pone.0192295 29420583 PMC5805292

[43] Discher BM, Maloney KM, Grainger DW, Hall SB. Effect of neutral lipids on coexisting phases in monolayers of pulmonary surfactant. Biophys Chem. 2002;101–102:333–45. 12488012 10.1016/s0301-4622(02)00191-6

[44] Echaide M, Autilio C, Arroyo R, Perez-Gil J. Restoring pulmonary surfactant membranes and films at the respiratory surface. Biochim Biophys Acta. 2017;1859(9 Pt B):1725–39. 10.1016/j.bbamem.2017.03.015 28341439

[45] Rudiger M, Tolle A, Meier W, Rustow B. Naturally derived commercial surfactants differ in composition of surfactant lipids and in surface viscosity. Am J Physiol Lung Cell Mol Physiol. 2005;288(2):L379–83. 10.1152/ajplung.00176.2004 15501950

[46] Fehrenbach H, Tews S, Fehrenbach A, Ochs M, Wittwer T, Wahlers T, et al Improved lung preservation relates to an increase in tubular myelin-associated surfactant protein A. Respir Res. 2005;6:60 10.1186/1465-9921-6-60 15969762 PMC1187923

[47] Hiansen JQ, Keating E, Aspros A, Yao LJ, Bosma KJ, Yamashita CM, et al Cholesterol-mediated surfactant dysfunction is mitigated by surfactant protein A. Biochim Biophys Acta. 2015;1848(3):813–20. 10.1016/j.bbamem.2014.12.009 25522687

[48] Rodriguez Capote K, McCormack FX, Possmayer F. Pulmonary surfactant protein-A (SP-A) restores the surface properties of surfactant after oxidation by a mechanism that requires the Cys6 interchain disulfide bond and the phospholipid binding domain. J Biol Chem. 2003;278(23):20461–74. 10.1074/jbc.M212697200 12600986

